# A novel serum microRNA signature to screen esophageal squamous cell carcinoma

**DOI:** 10.1002/cam4.973

**Published:** 2016-12-30

**Authors:** Zebo Huang, Lan Zhang, Danxia Zhu, Xia Shan, Xin Zhou, Lian‐wen Qi, Lirong Wu, Jun Zhu, Wenfang Cheng, Huo Zhang, Yan Chen, Wei Zhu, Tongshan Wang, Ping Liu

**Affiliations:** ^1^ Department of OncologyThe First Affiliated Hospital of Nanjing Medical University300 Guangzhou RoadNanjing210029China; ^2^ Department of OncologyThe Third Affiliated Hospital of Soochow University185 Juqian StreetChangzhou213003China; ^3^ Department of RespirationThe Affiliated Jiangning Hospital of Nanjing Medical University168 Gushan RoadNanjing210009China; ^4^ State Key Laboratory of Natural Medicines and Department of PharmacognosyChina Pharmaceutical UniversityNo. 24 Tongjia LaneNanjing210009China; ^5^ Department of Radiation OncologyJiangsu Cancer HospitalNo. 42 Bai Zi TingNanjing210009China; ^6^ Department of GastroenterologyThe First Affiliated Hospital of Nanjing Medical University300 Guangzhou RoadNanjing210029China; ^7^ Department of EmergencyThe First Affiliated Hospital of Nanjing Medical University300 Guangzhou RoadNanjing210029China; ^8^ Cancer Center of Nanjing Medical UniversityNanjing210029China

**Keywords:** Circulating microRNA, diagnostic biomarker, ESCC, qRT‐PCR

## Abstract

Circulating microRNAs (miRNAs) have been used as promising diagnostic biomarkers for esophageal squamous cell carcinoma (ESCC). We performed miRNA expression profiling using quantitative reverse transcription polymerase chain reaction (qRT‐PCR) based Exiqon panels from three ESCC pools and one normal control (NC) pool samples. Using qRT‐PCR, identified serum miRNAs were further confirmed in training (32 ESCC vs. 32 NCs) and testing stages (108 ESCC vs. 96 NCs). Consequently, five serum miRNAs (miR‐20b‐5p, miR‐28‐3p, miR‐192‐5p, miR‐223‐3p, and miR‐296‐5p) were significantly overexpressed in ESCC compared with NCs. The diagnostic value of the 5‐miRNA signature was validated by an external cohort (60 ESCC vs. 60 NCs). The areas under the receiver operating characteristic curve (ROC) of the 5‐miRNA signature were 0.753, 0.763, and 0.966 for the training, testing, and the external validation stages, respectively. The expression levels of the miRNAs were also determined in tissues, arterial serum, and exosomes. MiR‐20b‐5p, miR‐28‐3p, and miR‐192‐5p were significantly upregulated in ESCC tissues, while miR‐296‐5p was overexpressed in ESCC serum exosomes. In conclusion, we identified a 5‐miRNA signature in serum for the detection of ESCC.

## Introduction

Esophageal cancer is the fourth leading cause of cancer death in China, with estimated 477,900 newly diagnosed cases and 375,000 deaths in 2015 1. Esophageal squamous cell carcinoma (ESCC) is the predominant histological subtype of esophageal cancer, dominating almost 90% of esophageal cancer cases in China 2. Despite the improvements in diagnostic techniques, perioperative management, and chemoradiotherapy, ESCC still has a high probability of postoperative metastasis and recurrence, and remains a relatively low 5‐year survival rate 3. Most ESCC patients are diagnosed at a late stage due to lack of apparent symptoms and then miss the optimal time for operation. Thus, early examination and timely treatment can effectively optimize outcomes of ESCC patients. Existing routine detection approaches for ESCC diagnosis, including imaging examinations, endoscopy, and esophageal cytology, have been widely used in clinics. However, frequent imaging examinations, such as X‐ray and computed tomography (CT), might cause radiation risk to patients, whereas endoscopy is invasive and mostly determined by the experience of operators 4. Moreover, tumor markers such as squamous cell cancer antigen (SCC) and carcinoembryonic antigen (CEA) cannot show sufficient sensitivity and specificity 5. Taken together, it is important to identify novel and less invasive biomarkers for ESCC diagnosis.

MicroRNAs (miRNAs) are small (21–23 nucleotides) and highly conserved noncoding RNAs that negatively regulate gene expression post‐transcriptionally by binding the 3′‐untranslated region of target mRNAs, resulting in either mRNA degradation or translational repression 6. As some miRNAs are passively leaked or actively transported from cells, and circulating miRNAs could be stably detected in cell‐free body fluids, such as serum and plasma 7. Accumulating evidence has proved that circulating have emerged as potential diagnostic biomarkers in various cancers including ESCC 8, 9, 10. In the wake of developments in genomics, miRNA expression profiles have become more heated and accurate than mRNA expression profiles 11. However, ESCC serum miRNA profiles with relatively large sample are still rare to date. Therefore, in the present study, we focused on ESCC patients and conducted a four‐stage study to identify potential miRNAs for detecting ESCC by evaluating the expression of serum miRNAs based on qRT‐PCR. In addition, the identified miRNAs were confirmed in tissue samples and compared in arterial and peripheral serum samples. Exosomal miRNAs were also investigated to assess the potential form of the identified miRNAs in peripheral serum which might aid detection of ESCC.

## Materials and Methods

### Study design and clinical samples

A total of 200 histopathologically conformed ESCC patients and 188 healthy subjects were enrolled between 2013 and 2015. For the training and testing set, 140 ESCC patients and 128 healthy controls were recruited from the First Affiliated Hospital of Nanjing Medical University, while for external validation stage, we included another 60 ESCC patients and 60 healthy subjects from the Third Affiliated Hospital of Soochow University. All the procedures were approved by Institutional Review Boards of the two hospitals. Written informed consent was taken from each participant.

In this study, we carried out a four‐stage study for ESCC. In the initial screening stage, 30 peripheral serum samples from ESCC patients and 10 from NCs were randomly chosen and pooled as three ESCC samples and one NC sample (10 samples were pooled as 1 pool sample) for miRNA microarrays. Approximately 20–25 ng RNA isolated from each pool of serum samples was reverse transcribed to cDNA by using the miRCURY Locked Nucleic Acid (LNA^™^) Universal Reverse Transcription (RT) microRNA PCR, Polyadenylation, and cDNA synthesis kit (Exiqon miRNA qPCR panel, Vedbaek, Denmark) following the manufacturer's protocol. Microarrays were scanned on 7900HT real‐time PCR system (Applied Biosystems, Foster City, CA) with Exiqon miRCURY‐Ready‐to‐Use PCR‐Human‐panel‐I + II‐V1.M (Exiqon miRNA qPCR panel), which could detect 168 miRNAs in plasma/serum to identify differently expressed miRNAs. Melting curve analyses were performed at the end of the PCR cycles. Detectable miRNAs were those with a C_*t*_ < 37 and 5 C_*t*_ less than the negative control (no template control, NTC). An RNA spike‐in (UniSp6) and a DNA spike‐in (Sp3) were used as technical controls to evaluate if the technical performance of all samples is similar. The C_*t*_ values were normalized based on the average of the normalizer assays in the panel and this included miR‐191‐5p, miR‐423‐5p, miR‐425‐5p, and miR‐93‐5p. The formula used to calculate the normalized C_*t*_ values is: normalized C_*t*_ (ΔC_*t*_) = average C_*t*_ (assay) − average C_*t*_ (normalizer assays). The relative expression levels of miRNAs between ESCC patients and NCs were calculated using 2^−ΔΔC*t*^ method.

In the training stage, the differentially expressed miRNAs discovered via screening stage were confirmed using qRT‐PCR in 32 ESCC samples and 32 NCs. Then, the miRNAs identified by the training stage were further evaluated by qRT‐PCR in the testing stage in serum samples including 108 ESCC patients and 96 NCs. For the external validation set, we subjected 60 cases and 60 controls from another hospital to evaluate the diagnostic value of the 5‐miRNA signature in ESCC. And the selected miRNAs were further verified in 36 pairs of formalin‐fixed paraffin‐embedded (FFPE) ESCC tissue specimens and adjacent nontumor tissues from surgery patients. Arterial blood samples were collected from 10 ESCC patients and were identified to compare the difference of miRNAs between peripheral and arterial serum. In addition, exosomal miRNAs from 32 patients and NCs were analyzed to investigate the potential form of the miRNAs in the peripheral serum.

Whole blood sample of each participant was collected and subjected to centrifuge at 3000 g for 10 min within 12 h after collection. Then, cell‐free serum was further resolved by centrifugation at 10,000 g for 2 min to guarantee complete removal of cell debris. The serum sample was stored in an RNase‐free eppendorf tube at −80°C until use.

### RNA extraction

RNA was isolated from 200 *μ*L serum using mirVana Paris Kit (Ambion, Austin, TX) according to the manufacturer's protocol. Total RNA was extracted from FFPE specimens using the High Pure FFPE RNA Micro Kit (Ambion), as previously described. RNA was eluted with 100 *μ*L of RNase‐free water and stored at −80°C for further use. The ultraviolet spectrophotometer was used to evaluate the concentration and purity of the total RNA. The concentration of serum RNA ranged from 15.26 to 50.19 ng/*μ*L.

### Isolation of exosomes

ExoQuick Exosome Precipitation Solution (System Biosciences, Mountain View, CA) was used to isolate exosomes from serum according to the manufacturer's protocol. Briefly, 200 *μ*L serum was mixed with 50 *μ*L ExoQuick exosome precipitation solution and then kept at 4°C for 30 min, followed by centrifugation at 12,000 g for 2 min. After the supernatants were removed, the exosome pellets were retained for further RNA extraction.

### Quantitative reverse transcription polymerase chain reaction (qRT‐PCR)

The amplification of miRNA was performed using the specific primers of reverse transcription (RT) and polymerase chain reaction (PCR) from Bulge‐Loop^™^ miRNA qRT‐PCR Primer Set (RiboBio, Guangzhou, China) as previously described. The quantification of PCR product was evaluated by the level of fluorescence in emitted by SYBR Green (SYBR^®^ Premix Ex Taq^™^ II, TaKaRa). RT and PCR were performed as previously described. RT reactions were carried out at 42°C for 60 min followed by 70°C for 10 min. The qRT‐PCR was run on a LightCycler^®^ 480 Real‐Time PCR System (Roche Diagnostics, Mannheim, Germany) in 384‐well plates at 95°C for 20 sec, followed by 40 cycles of 95°C for 10 sec, 60°C for 20 sec, and then 70°C for 10 sec. The melting analysis was added finally to evaluate the specificity of PCR products. The expression of miRNAs in serum was determined using the 2^−∆Ct^ method relative to the endogenous reference miRNA (miR‐16), ΔC_*t*_ = Ct_miRNA_ − Ct_miR‐16_ 12. The relative levels of miRNAs in tissue specimens and exosomes were calculated using the comparative 2^−ΔΔC*t*^ method relative to *RNU6B* (*U6*) and miR‐16, respectively.

### Statistical analysis

Mann–Whitney test was used to analyze differential miRNAs expression between ESCC patients and NCs. The association between miRNAs and the clinical characteristics was evaluated by the one‐way ANOVA or chi‐square test. Receiver operating characteristic (ROC) curves and the area under the ROC curve (AUC) were used to estimate the diagnostic value of the candidate miRNAs for ESCC. Logistic regression model for ESCC prediction was applied on the data from the training and validation stages. All the statistical analyses were performed using SPSS software (version 20.0, IBM, North Castle, NY). A two‐sided *P* < 0.05 was defined as statistical significance.

## Results

### Characteristics of subjects

**Figure 1:**
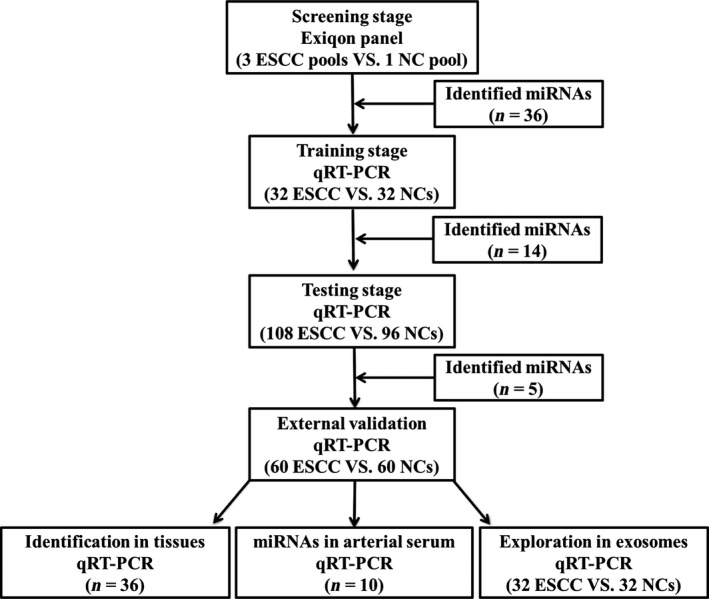
The flowchart of the experiment design. ESCC, esophageal squamous cell carcinoma; NC, normal control.

A total of 388 subjects, including 200 ESCC patients and 188 NCs, were included in our study. The flowchart of the experiment (Fig. 1) showed that our study was divided into three stages after the screening stage: the training stage, the testing stage, and the external validation stage. As shown in Table**** 1, there was no significant difference in the distribution of age or gender between ESCC patients and NCs in any of the three cohorts (*P *> 0.05).

**Table 1:** Clinical characteristics of 200 esophageal squamous cell carcinoma patients

Variables	Training phase (*n* = 32)	Testing phase (*n* = 108)	External validation stage (*n* = 60)
Age (mean ± SD)	67.62 ± 10.21	65.04 ± 10.55	63.11 ± 7.12
Gender (%)			
Male	20 (62.5)	65 (60.2)	37 (61.7)
Female	12 (37.5)	43 (39.8)	23 (38.3)
Tumor size (%)			
<5 cm	19 (59.4)	68 (63.0)	35 (58.3
≥5 cm	13 (40.6)	40 (37.0)	25 (41.7)
Tumor location (%)			
Upper	8 (25.0)	26 (24.1)	15 (25.0)
Middle	18 (56.2)	63 (58.3)	37 (61.7)
Lower	6 (18.8)	19 (17.6)	8 (13.3)
Differentiation grade (%)			
Well/moderate	22 (68.8)	70 (64.8)	41 (68.3)
Poorly	10 (31.2)	38 (35.2)	19 (31.7)
TNM stage (%)			
I	8 (25.0)	35 (32.4)	23 (38.3)
II	6 (18.8)	17 (15.7)	15 (25.0)
III	14 (43.7)	43 (39.8)	20 (33.3)
IV	4 (12.5)	13 (12.1)	2 (3.40)

### MiRNA profiling in the screening stage

To identify candidate serum miRNAs for ESCC diagnosis, the Exiqon miRCURY‐Ready‐to‐Use PCR‐Human‐panel‐I + II‐V1.M was conducted based on the qRT‐PCR platform. A total of 168 miRNAs were initially screened in three ESCC and one NC pooled serum samples. Candidate miRNAs with a cycle threshold (C_*t*_) value <37 and 5 lower than negative control (NTC) in the panel were further selected for analysis. By this standard, 33 upregulated miRNAs and 3 downregulated miRNAs showed at least a 1.5‐fold altered expression in 3 ESCC pooled samples compared to 1 NC pooled sample, respectively (Table S1). Then, these dysregulated miRNAs were selected to further validation stages.

### Evaluation of candidate miRNAs by qRT‐PCR

**Figure 2:**
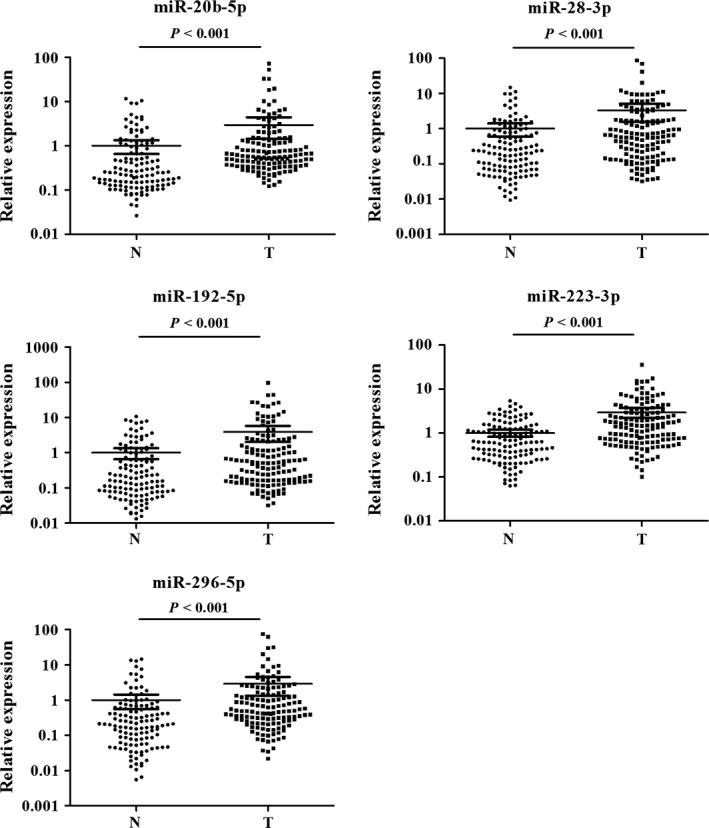
Expression levels of the five miRNAs in the serum of 140 esophageal squamous cell carcinoma patients and 128 NCs (in the training and testing stages). N, normal controls; T, tumor. Horizontal line: mean with 95% CI.

The 36 miRNAs identified through the screening stage were validated in the training stage including 32 ESCC patients and 32 NCs using qRT‐PCR analysis. A total of 14 differentially expressed miRNAs were identified and then were confirmed in the testing stage with a larger sample set. Consequently, five miRNAs (miR‐20b‐5p, miR‐28‐3p, miR‐192‐5p, miR‐223‐3p, and miR‐296‐5p) showed significantly elevated expression levels in ESCC serum, consistent with those in the training stage (Fig. S1). Additionally, all the five miRNAs were significantly upregulated in serum of ESCC patients when the two stages were combined (Fig. 2), suggesting that the five identified miRNAs in peripheral serum might act as potential diagnostic markers for ESCC patients.

### Diagnostic value of miRNAs in serum

**Figure 3:**
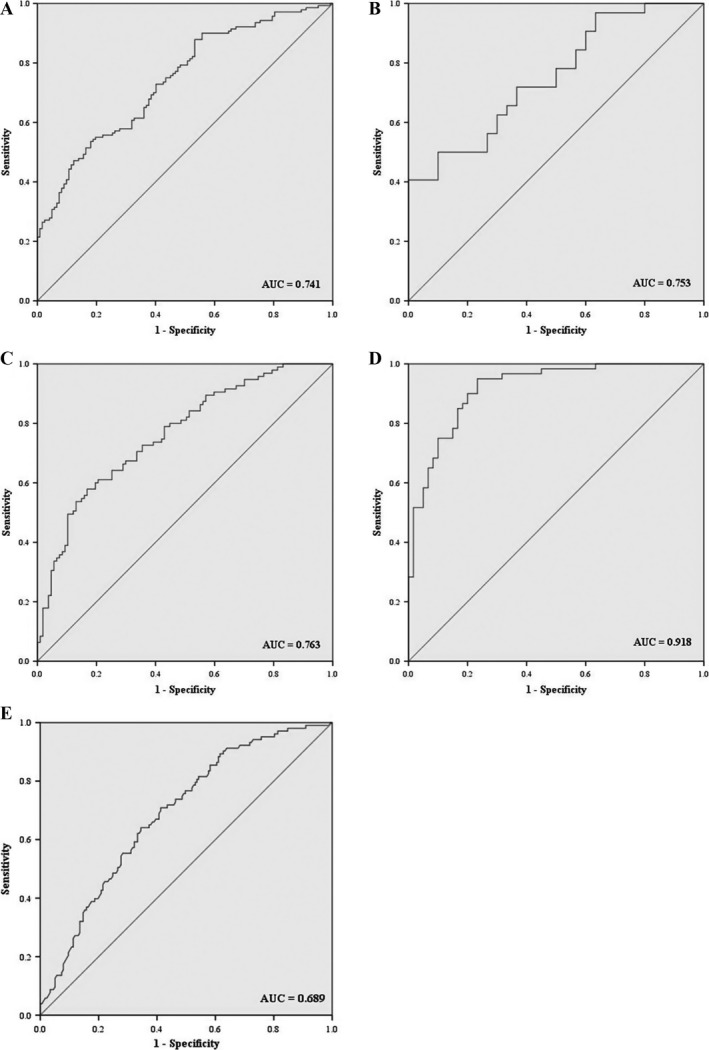
Receiver operating characteristic (ROC) curves for the 5‐miRNA signature to discriminate ESCC patients from NCs. (A) The combined two phases of training and testing stages (140 ESCC vs. 128 NCs); (B) training stage (32 ESCC vs. 32 NCs); (C) testing stage (108 ESCC vs. 96 NCs); (D) external cohort (60 ESCC vs. 60 NCs); (E) patients with stage I and II (104 ESCC vs. 188 NCs). AUC, areas under the curve; ESCC, esophageal squamous cell carcinoma.

To assess the diagnostic value of the five miRNAs in discriminating ESCC patients from NCs, the optimal cutoff values for miR‐20b‐5p, miR‐28‐3p, miR‐192‐5p, miR‐223‐3p, and miR‐296‐5p were determined according to ROC curves for each miRNA in the combined cohorts from the training and testing stages. The AUCs were 0.731, 0.656, 0.662, 0.736, and 0.689 for miR‐20b‐5p, miR‐28‐3p, miR‐192‐5p, miR‐223‐3p, and miR‐296‐5p, respectively (Fig. S2). Furthermore, when the five miRNAs were combined together as a panel, it showed a higher accuracy than any individual miRNA in discriminating ESCC patients from NCs (AUC: 0.741; 95% CI: 0.682–0.800; Fig. 3A). Meanwhile, the diagnostic value of the 5‐miRNA panel was also assessed in the training and testing stages separately and the AUCs were 0.753 (95% CI: 0.635–0.872; Fig. 3B) and 0.763 (95% CI: 0.698–0.828; Fig. 3C), respectively.

To further assess the diagnostic capacity of the 5‐miRNA panel for diagnosing ESCC, an external cohort including 60 ESCC patients and 60 NCs from a different medical center was validated. As compared with NCs, the five miRNAs were overexpressed in ESCC patients, consistent with the results in the training and the testing stages (Fig. S3). In the external cohort, this panel could also accurately discriminate ESCC patients from NCs with AUC of 0.918 (95% CI: 0.870–0.966) (Fig. 3D).

We further analyzed the relationship of the five miRNAs in serum with tumor stage in all 200 ESCC patients. All the five miRNAs showed higher expression in patients with stage III + IV compared to those with stage I + II, but only miR‐20b‐5p was statistically significant (Fig. S4). Furthermore, the diagnostic value of the 5‐miRNA panel was evaluated in 104 patients with stage I + II and all NCs, and the AUC was 0.689 (95% CI: 0.627–0.751) (Fig. 3E).

### Evaluation of miRNAs in tissue samples

**Figure 4:**
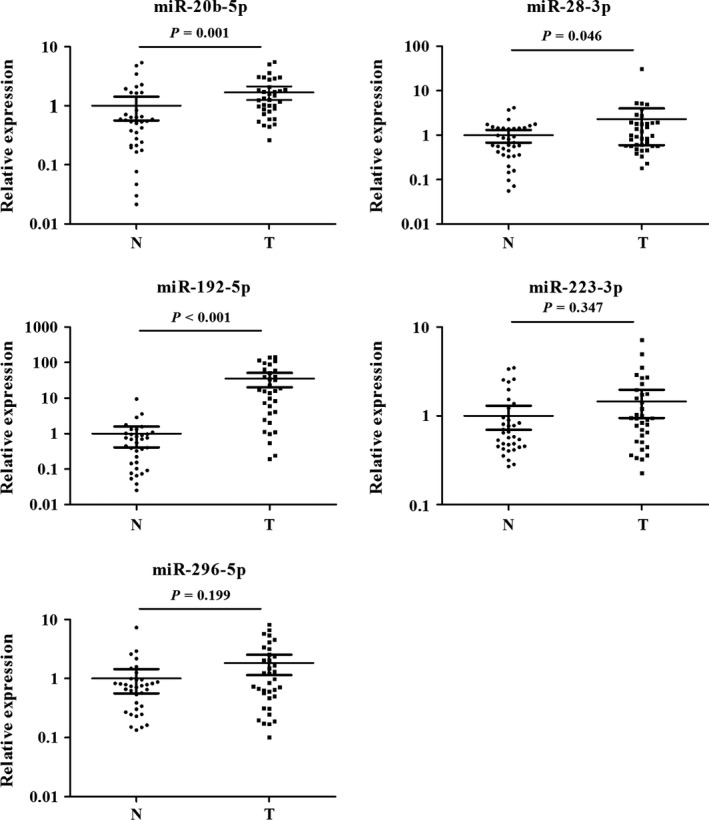
Expression of the five miRNAs in the tumor tissues of 36 ESCC patients and 36 NCs. N, normal controls; T, tumor. Horizontal line: mean with 95% CI. ESCC, esophageal squamous cell carcinoma.

To explore the consistency of the identified miRNAs in serum and tissue of ESCC patients, we examined the expression levels of the five miRNAs in an additional of 36 pairs of tissue samples. All the five miRNAs had higher expression in tumor samples than in normal tissues, but only miR‐223‐3p and miR‐296‐5p did not show statistically significant difference (Fig. 4).

### Comparison of miRNAs in peripheral and arterial serum

Next, we explored the expression levels of the five miRNAs in 10 arterial serum samples and 10 matched peripheral serum samples to identify the differences in miRNA expression between peripheral and arterial serum. As a result, the expression levels of the six miRNAs have no significant differentiation between peripheral and arterial serum (Fig. S5).

### Exploration of miRNAs in serum exosomes

**Figure 5:**
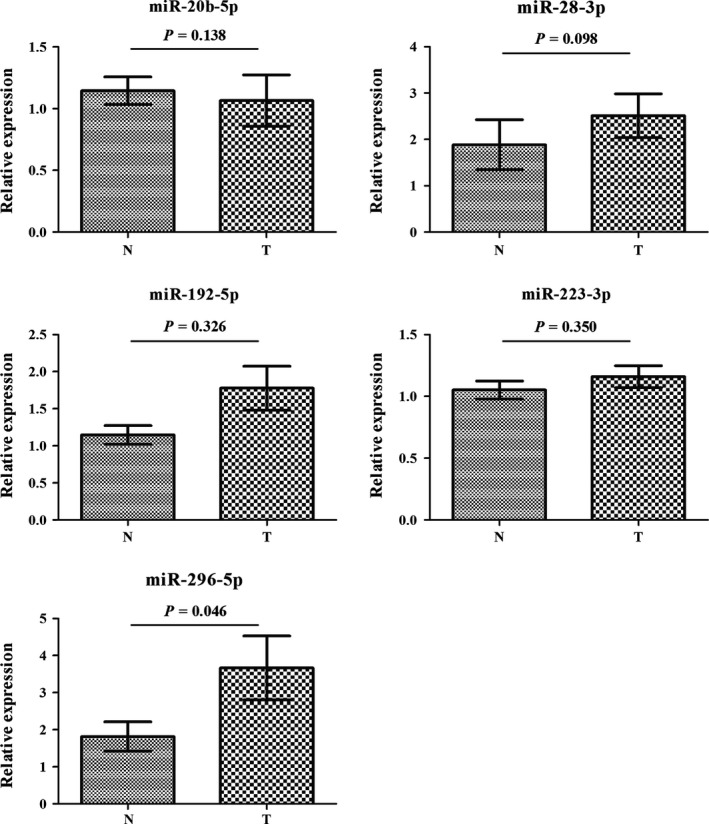
Expression of the five miRNAs in the serum exosomes of 28 ESCC patients and 28 NCs. Error bar: standard error. N, normal controls; T, tumor; ESCC, esophageal squamous cell carcinoma.

For further research, exosomal miRNAs extracted from 28 ESCC and 28 NC serum samples were explored to assess the potential form of the identified miRNAs in peripheral serum. Compared to NCs, miR‐28‐3p, miR‐192‐5p, miR‐223‐3p, and miR‐296‐5p were upregulated in ESCC serum exosomes, but only miR‐296‐5p was with statistical significance (Fig. 5).

## Discussion

Recently, circulating miRNAs in body fluids have emerged as potential diagnostic biomarkers for various cancers 13. Microarray profiling is used to figure out specific miRNA expression profiles for cancer detection. In this current study, we designed a four‐stage study to identify serum‐based miRNAs for ESCC diagnosis. Exiqon miRNA qPCR panels, involved in our previous study 9, were applied to analyze differential expression profiling of serum miRNAs in three ESCC and one NC pooled samples in the screening stage. Next, we assessed the candidate miRNAs by qRT‐PCR in the validation stages. Without doubt, the option of appropriate reference miRNAs for data normalization was the point of qRT‐PCR. Reference miRNAs should express stably in circulation and show little difference between cancer and normal samples. However, as far as we know there is still no consensus reference gene for miRNA quantification. In our previous experimental work, miR‐16‐5p was discovered to be stable and detectable in serum. In addition, miR‐16‐5p has been selected as reference in accumulating studies and is relatively stable in the circulation 14, 15. Hence, we chose miR‐16‐5p as the reference miRNA. Through the training and the testing stage, miR‐20b‐5p, miR‐28‐3p, miR‐192‐5p, miR‐223‐3p, and miR‐296‐5p were identified to be significantly upregulated in ESCC serum, and the expression level of miR‐20b‐5p was significantly higher in patients with advanced stage (III and IV). The external validation stage from another medical center was conducted to verify the reliability and reproducibility of the diagnostic value of the 5‐miRNA signature. As blood flows from arterial to venous circulation, we speculated that circulating miRNAs released from tumor cells might present higher expression levels in arterial blood than those in peripheral serum. But there were no significant differences in our result. Additionally, miR‐20b‐5p, miR‐28‐3p, and miR‐192‐5p showed consistently high expression in ESCC tissues, while miR‐296‐5p was upregulated in ESCC serum exosomes.

For the similarities among a variety of tumors, the identified five miRNAs might overexpress in serum of patients with some other tumors. Thus, we compared serum‐based miRNA profiles from other cancers with the 5‐miRNA signature. It seemed that miR‐192‐5p and miR‐223‐3p were widely studied. It is reported that miR‐192‐5p was upregulated in serum from patients with hepatocellular carcinoma 16 and pancreatic ductal adenocarcinoma 17, while miR‐223‐3p overexpressed in colorectal cancer 18 and gastric cancer 19. However, there are few reports about serum‐based miRNA profiles containing the other three miRNAs in cancers. In our other ongoing experiments, we found that miR‐296‐5p was upregulated in gastric cancer and thyroid cancer. Although the 5‐miRNA signature had certain specificity in ESCC, the similarities and differences in miRNAs among different cancers need further study.

Certainly, plenty of current studies focus on the mechanisms of miRNAs in the development and progression of cancer. Among the five miRNAs identified in our study, circulating miR‐20b‐5p was discovered for the first time to be a valuable biomarker of ESCC in our study. MiR‐20b‐5p is one component of the miR‐106a‐363 cluster, which like miR‐17‐92 cluster and miR‐106b‐25 cluster belongs to the miR‐17 family 20, 21. PTEN, a tumor suppressor, was identified to be a target of miR‐20b‐5p 22. These findings indicated that miR‐20b‐5p might be an oncogene in ESCC. And it was found to be upregulated in tissues of breast cancer patients with brain metastasis 23. As for miR‐28‐3p, it was found markedly upregulated in esophageal cancer tissues 24. However, little research has been done in cancer about the miRNA, and it seemed that miR‐28‐3p was associated with endocrine disorders, such as type 2 diabetes 25 and childhood obesity 26. The potential mechanism of miR‐28‐3p in ESCC is worthy of further research. Instead, miR‐192‐5p has been extensively studied in numerous tumors. In ESCC, miR‐192‐5p was a potential biomarker in prediction of multimodality therapy response 27. There were several targets of miR‐192‐5p discovered in some cancers. For instance, retinoblastoma 1 (RB1) was a direct target of miR‐192‐5p, which induced cell apoptosis through the caspase pathway 28. And miR‐192‐5p targeted smad‐interacting protein 1 (SIP1) to alter cell cycle regulatory genes in PDAC 17. When it comes specifically to ESCC, the overexpression of miR‐192 could inhibit cells apoptosis and promote ESCC cells proliferation by targeting 3′‐UTR of *Bim* gene directly 29. However, a recent research which performed systems‐based miRNA analyses of large‐scale patient data sets along with in vitro and in vivo experiments reported that miR‐192 was a key regulator of angiogenesis 30. Through regulating EGR1 and HOXB9, miR‐192‐5p downregulated the angiogenic pathways in cancer cells. Although this antiangiogenic and antitumor effect might be contrary to our results, this precisely indicated the duplicity of miRNAs in cancer. MiR‐223‐3p, another well‐studied miRNA, was found overexpressed in serum and tissue samples and could be used as a potential diagnostic and prognostic marker in ESCC patients 10, 31, 32. These results were consistent with our findings. Kurashige et al. suggested that FBXW7 was a functional downstream target of miR‐223‐3p, and the overexpression of miR‐223‐3p led to the reduced expression of FBXW7, which gave rise to the abnormal accumulation of c‐Myc and c‐Jun proteins 31. As an angiogenesis‐related angiomiR, miR‐296‐5p promoted angiogenesis in tumors by directly targeting the hepatocyte growth factor‐regulated tyrosine kinase substrate (HGS), and thereby reducing HGS‐mediated degradation of VEGFR2 and PDGFR‐β 33. It is reported that miR‐296‐5p overexpressed in both carcinoma in situ and ESCC tissues, and in vitro experiment showed that inhibition of miR‐296‐5p increased sensitivity to chemotherapeutic drugs in esophageal carcinoma cell lines 34. Of course, further research should be conducted to uncover the detail mechanism for the deregulation of these miRNAs in ESCC formation and development.

It is well known that disease‐related miRNAs could be shed from cells or tissues and be present in circulation 13. We assessed the expression of the five identified serum miRNAs in ESCC tissues and paired adjacent nontumor tissues. As a result, all the five miRNAs overexpressed in tumor samples than in normal tissues, though miR‐223‐3p and miR‐296‐5p did not show statistically significant difference. The subtle differences between serum and tissue might be caused by the different degradation and absorption efficiency of miRNAs in circulation and tissue. And we suggested that the miRNAs in circulation reflected the whole status of ESCC, but miRNAs in tissue only reflected the part changes of local tumor. In addition, circulating miRNAs were found to be originated from the cell‐derived exosomes which could protect miRNAs against degradation by ribonuclease. Exosomes are small (40–100 nm) membrane vesicles secreted by most types of cell including cancer cell. Theses vesicles could protect the miRNAs, mRNAs, and proteins from degradation by various enzymes outside and transfer messages among cells that make them the most important intercellular communicators 35. We further explored serum exosomal miRNAs to better understand the potential form of miRNAs in serum. It turned out that miR‐296‐5p was significantly upregulated in serum exosomes from ESCC patients, while the expression levels of exosomal miR‐28‐3p, miR‐192‐5p, and miR‐223‐3p had the trend of increasing in ESCC patients. Reportedly, serum exosomal miR‐223‐3p was found to be upregulated in primary CRC and esophageal adenocarcinoma patients 36, 37. An excellent study to characterize circulating miRNA complexes in human plasma and serum revealed that circulating miRNAs not only cofractionated with vesicles like exosomes, but also copurified with the Ago2 ribonucleoprotein complex 38. Therefore, the mechanisms of the generation and existent forms of circulating miRNAs still needs more research.

In conclusion, we conducted a multistep investigation to identify serum miRNAs ESCC detection, and a 5‐miRNA signature was identified as noninvasive biomarker. Further study needs to focus on larger sample validation and the mechanisms of the five miRNAs in ESCC.

## Conflicts of Interest

None declared.

## Supporting information


**Figure S1.** Expression levels of the five miRNAs in the serum of ESCC patients and NCs in the training and testing stages, respectively.
**Figure S2.** ROC curve analyses of each miRNA to discriminate ESCC patients from NCs in the combined two stages.
**Figure S3.** Expression levels of the five miRNAs in the serum of ESCC patients and NCs in the external cohort.
**Figure S4.** Comparison of the five miRNAs in serum of ESCC patients diagnosed with early (I + II) and late (III + IV) stage disease.
**Figure S5.** Comparison of the six miRNAs in 10 arterial serum samples and matched peripheral serum samples.
**Table S1.** Differently expressed miRNAs in the screening phase.
