# Supplementary material for: A novel serum microRNA signature to screen esophageal squamous cell carcinoma
Source: Cancer Med. 2016 Dec 30;6(1):109–19. doi: 10.1002/cam4.973 (PMC5269712; doi:10.1002/cam4.973)

**Supporting Information**

| Table S1. Differently expressed miRNAs in the screening phase | | | |  |  |
| --- | --- | --- | --- | --- | --- |
| **MiRNA** | | **Fold change** | | | **Mean fold** |
|  |  | **Pool 1** | **Pool 2** | **Pool 3** |  |
| let-7b-3p | | 1.60 | 1.60 | 2.04 | 1.75 |
| **miR-106a-5p** | | 1.73 | 1.68 | 2.04 | 1.82 |
| miR-141-3p | | 4.52 | 5.52 | 7.84 | 5.96 |
| miR-144-5p | | 2.17 | 2.15 | 2.45 | 2.26 |
| miR-146b-5p | | 1.72 | 2.48 | 3.05 | 2.42 |
| **miR-154-5p** | | 1.68 | 1.82 | 2.96 | 2.16 |
| **miR-17-5p** | | 2.36 | 2.48 | 5.98 | 3.61 |
| miR-18a-5p | | 2.69 | 2.28 | 3.64 | 2.87 |
| miR-18b-5p | | 2.46 | 3.24 | 3.22 | 2.98 |
| miR-190a | | 2.61 | 1.88 | 1.95 | 2.15 |
| **miR-192-5p** | | 2.02 | 2.10 | 1.79 | 1.97 |
| miR-200a-3p | | 1.88 | 1.62 | 4.80 | 2.77 |
| miR-205-5p | | 2.68 | 4.47 | 4.51 | 3.89 |
| **miR-20b-5p** | | 1.88 | 1.51 | 2.31 | 1.90 |
| **miR-21-5p** | | 1.83 | 1.52 | 1.89 | 1.75 |
| **miR-223-3p** | | 1.52 | 1.69 | 1.61 | 1.61 |
| **miR-25-3p** | | 1.54 | 1.52 | 1.56 | 1.54 |
| **miR-28-3p** | | 12.76 | 10.66 | 31.54 | 18.32 |
| miR-28-5p | | 1.72 | 2.34 | 2.57 | 2.21 |
| **miR-296-5p** | | 2.68 | 4.76 | 6.41 | 4.62 |
| miR-29a-5p | | 1.53 | 2.01 | 3.94 | 2.49 |
| miR-32-5p | | 1.52 | 1.71 | 1.85 | 1.69 |
| **miR-34a-5p** | | 2.59 | 2.64 | 2.79 | 2.67 |
| miR-374a-5p | | 1.76 | 1.72 | 2.94 | 2.14 |
| miR-382-5p | | 1.51 | 1.62 | 2.00 | 1.71 |
| miR-424-5p | | 1.73 | 1.59 | 1.67 | 1.66 |
| **miR-486-5p** | | 1.51 | 1.59 | 2.14 | 1.75 |
| **miR-495-3p** | | 2.63 | 1.96 | 3.37 | 2.66 |
| **miR-497-5p** | | 2.35 | 2.71 | 1.98 | 2.35 |
| miR-501-3p | | 1.78 | 1.81 | 1.73 | 1.77 |
| miR-502-3p | | 2.13 | 2.57 | 1.78 | 2.16 |
| miR-551b-3p | | 2.16 | 2.24 | 4.96 | 3.12 |
| miR-584-5p | | 1.79 | 2.23 | 2.17 | 2.06 |
| miR-151a-3p | | -3.50 | -2.69 | -2.79 | -2.99 |
| miR-409-3p | | -10.72 | -8.46 | -5.87 | -8.35 |
| miR-543 | | -4.29 | -4.91 | -4.40 | -4.53 |

**Note:** miRNAs in bold and underline were identified after the training stage.

**Figure S1** Expression levels of the five miRNAs in the serum of ESCC patients and NCs in the training and testing stages, respectively. N: normal controls; T: tumor. The whiskers of box plots: Tukey.

**
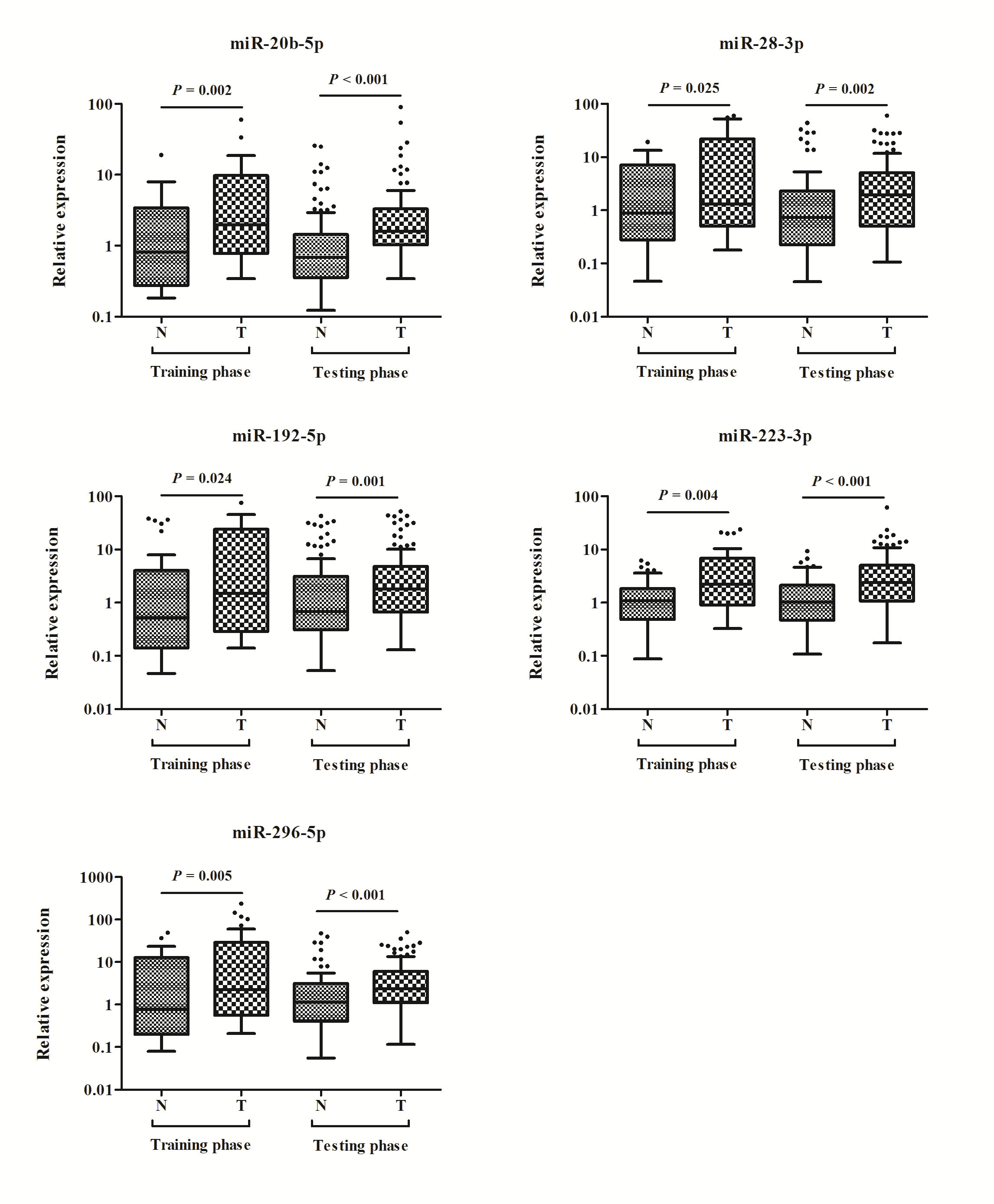
Figure S2** ROC curve analyses of each miRNA to discriminate ESCC patients from NCs in the combined two stages.


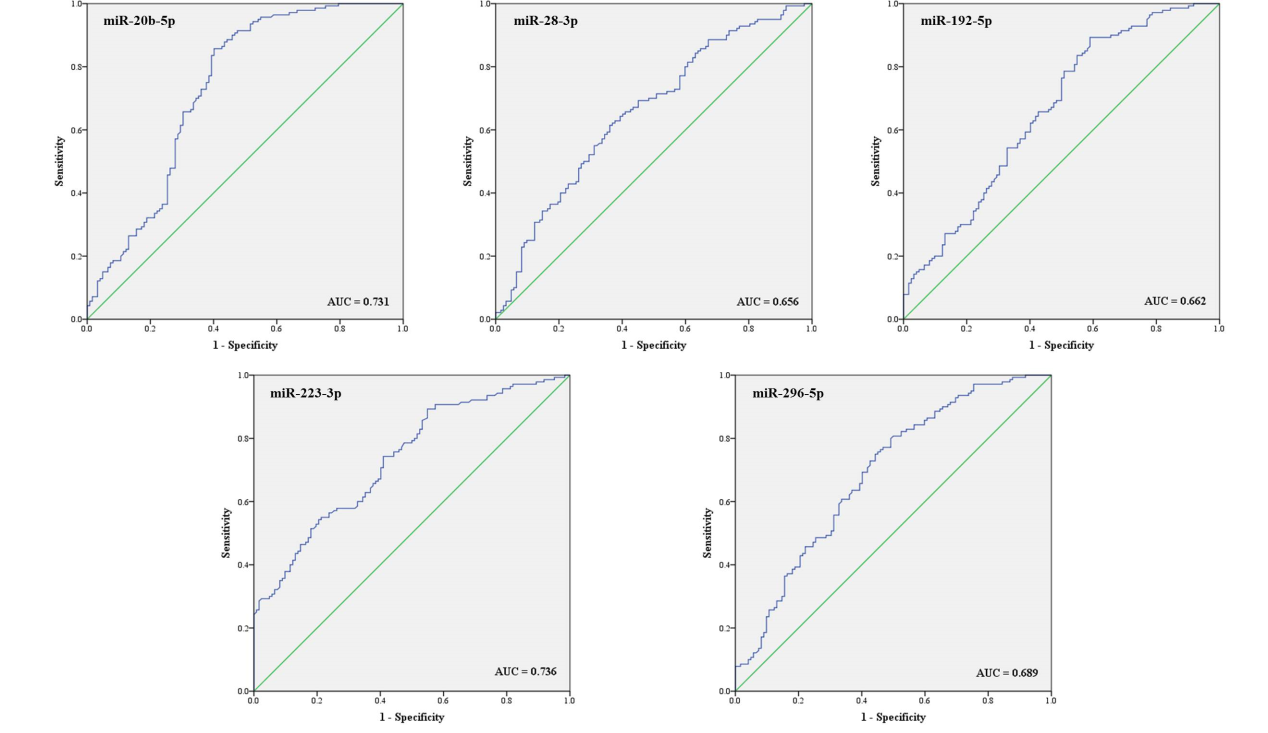


**Figure S3** Expression levels of the five miRNAs in the serum of ESCC patients and NCs in the external cohort. N: normal controls; T: tumor. Horizontal line: mean with 95% CI.


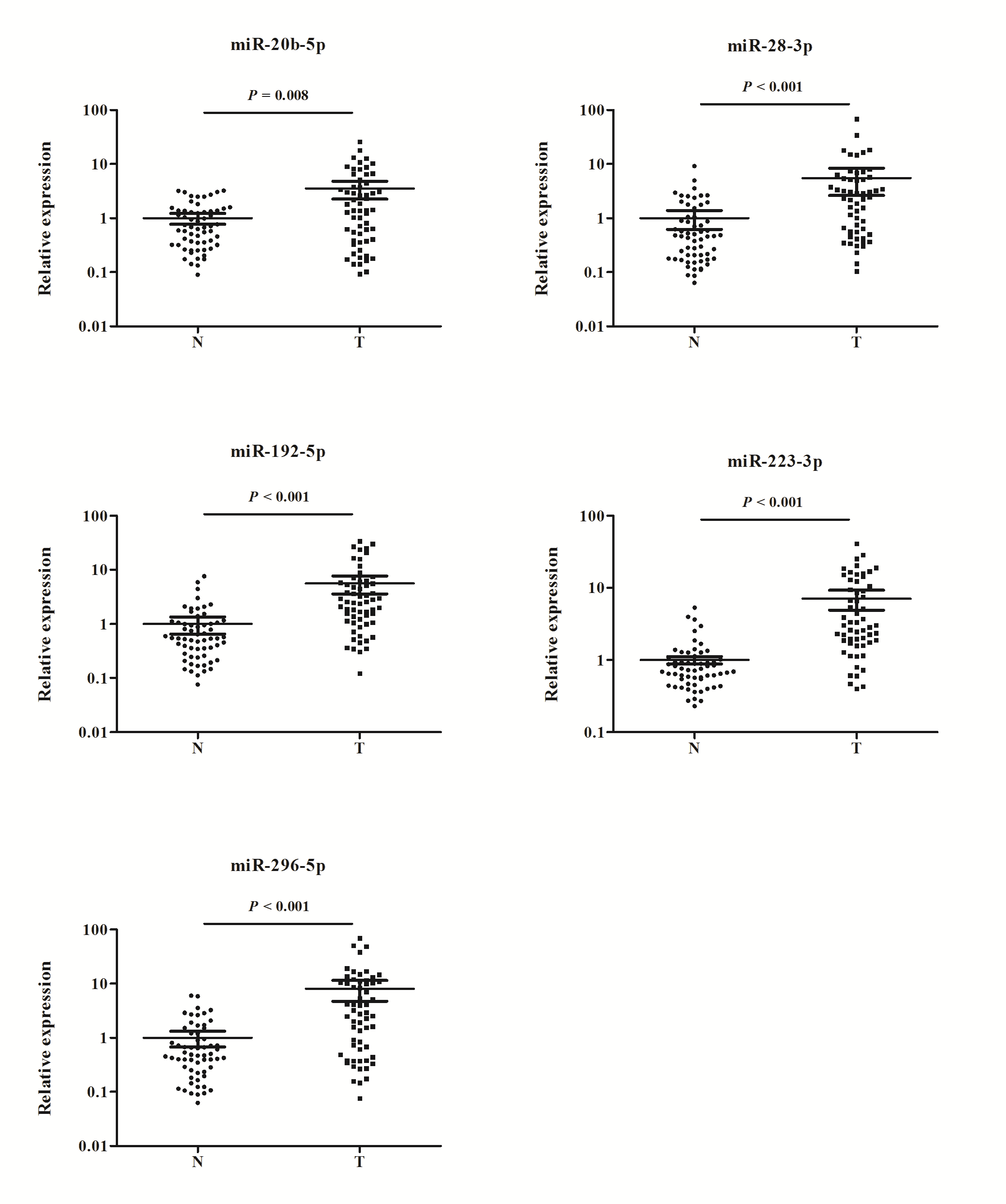


**Figure S4** Comparison of the five miRNAs in serum of ESCC patients diagnosed with early (I+ II) and late (III+ IV) stage disease. Error bar: standard error.


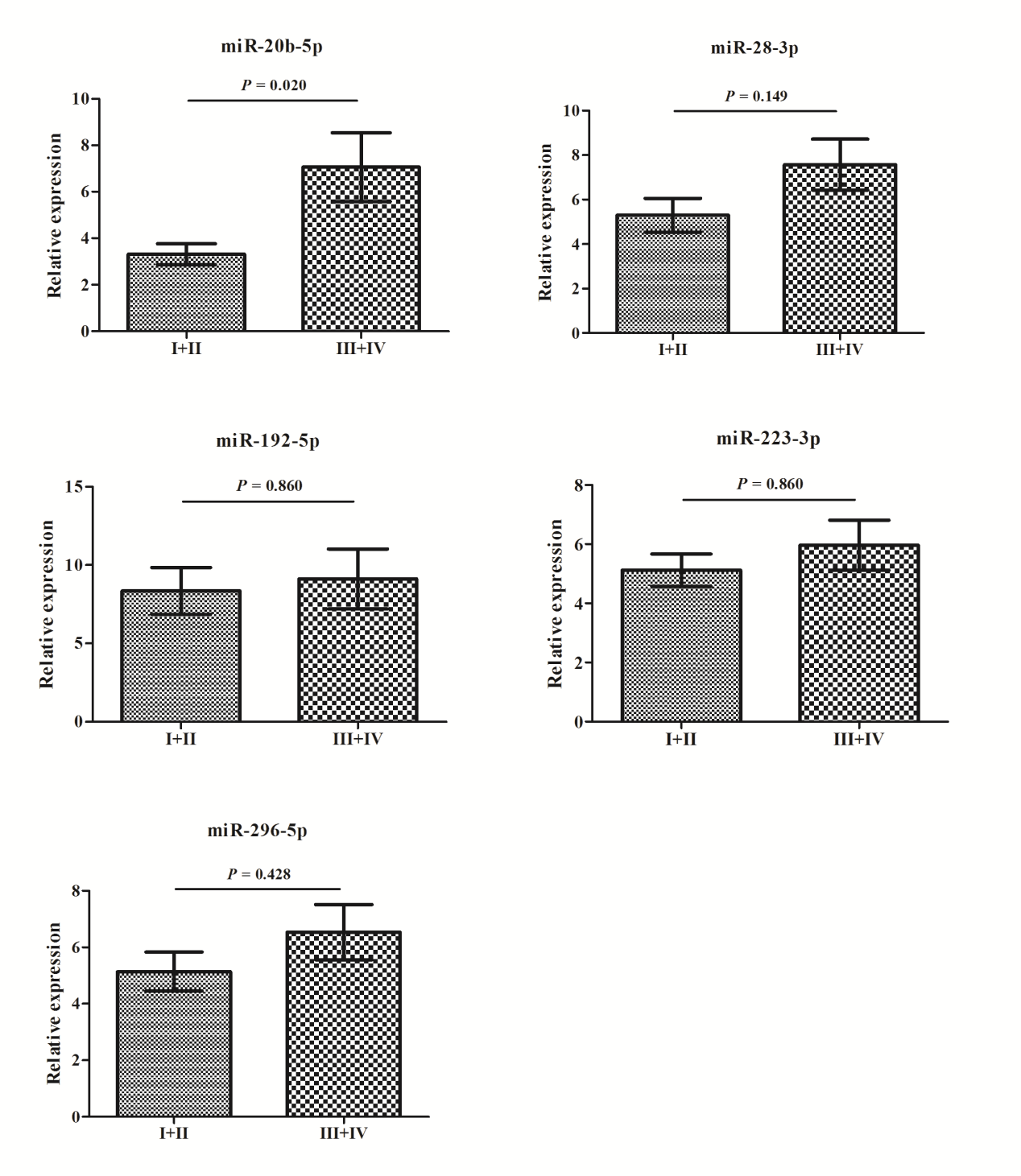
**Figure S****5** Comparison of the six miRNAs in 10 arterial serum samples and matched peripheral serum samples.


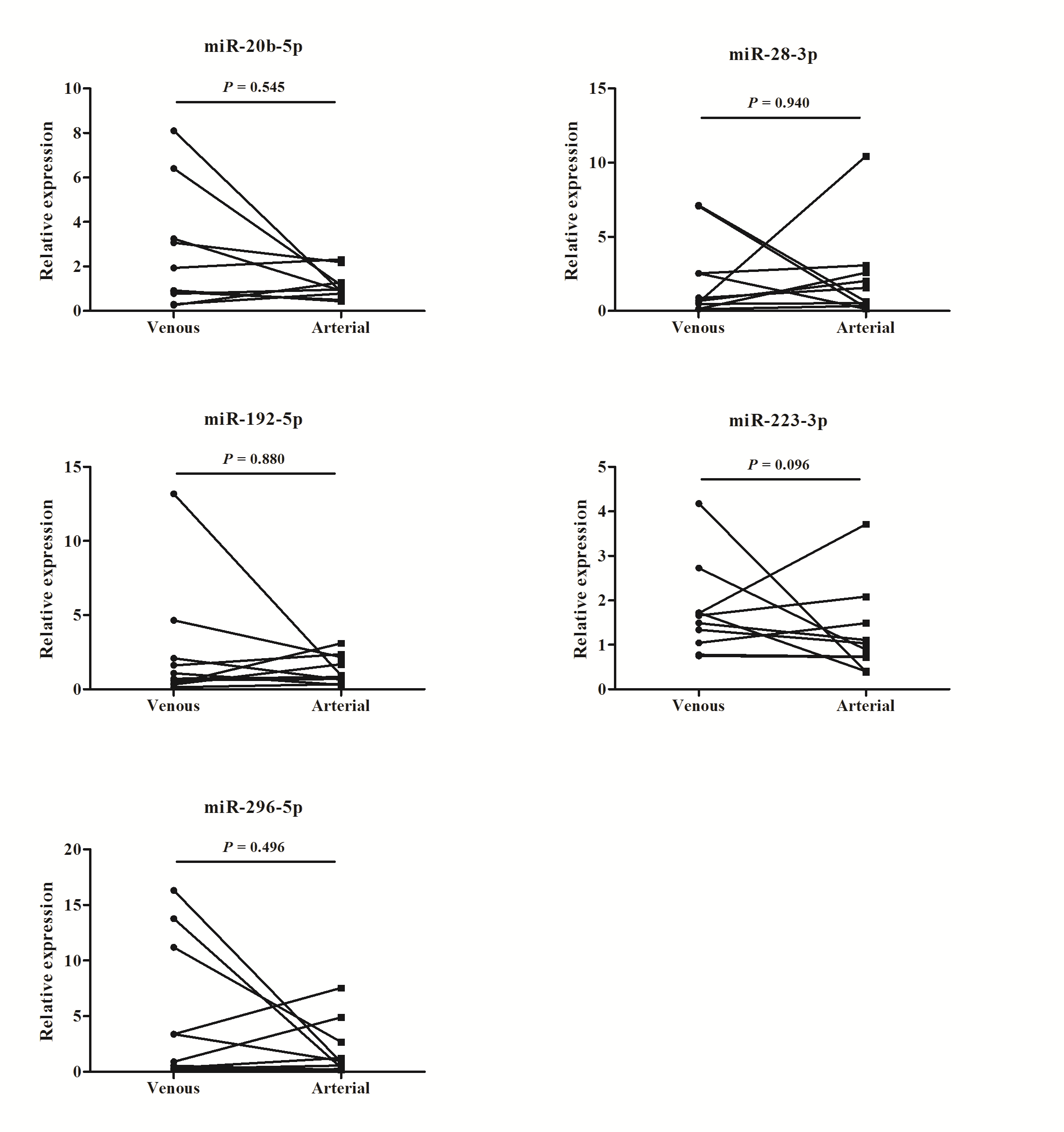

Supplement: Supplementary file 1 — Figure S1. Expression levels of the five miRNAs in the serum of ESCC patients and NCs in the training and testing stages, respectively. Figure S2. ROC curve analyses of each miRNA to discriminate ESCC patients from NCs in the combined two stages. Figure S3. Expression levels of the five miRNAs in the serum of ESCC patients and NCs in the external cohort. Figure S4. Comparison of the five miRNAs in serum of ESCC patients diagnosed with early (I + II) and late (III + IV) stage disease. Figure S5. Comparison of the six miRNAs in 10 arterial serum samples and matched peripheral serum samples. Table S1. Differently expressed miRNAs in the screening phase. [file CAM4-6-109-s001.docx]
